# Surfactant-Free Electrosprayed Alginate Beads for Oral Delivery of Hydrophobic Compounds

**DOI:** 10.3390/polym17152098

**Published:** 2025-07-30

**Authors:** Hye-Seon Jeong, Hyo-Jin Kim, Sung-Min Kang, Chang-Hyung Choi

**Affiliations:** 1 School of Chemical Engineering, Yeungnam University, 280, Daehak-ro, Gyeongsan 38541, Republic of Korea; 2 Department of Green Chemical Engineering, Sangmyung University, Cheonan 31066, Republic of Korea

**Keywords:** alginate hydrogel, electrospray, surfactant-free, hydrophobic compound delivery, controlled release, oral drug delivery

## Abstract

Oral delivery of hydrophobic compounds remains challenging due to their poor aqueous solubility and the potential toxicity associated with conventional surfactant-based emulsions. To address these issues, we present a surfactant-free encapsulation strategy using electrosprayed alginate hydrogel beads for the stable and controlled delivery of hydrophobic oils. Hydrophobic compounds were dispersed in high-viscosity alginate solutions without surfactants via ultrasonication, forming kinetically stable oil-in-water dispersions. These mixtures were electrosprayed into calcium chloride baths, yielding monodisperse hydrogel beads. Higher alginate concentrations improved droplet sphericity and suppressed phase separation by enhancing matrix viscosity. The resulting beads exhibited stimuli-responsive degradation and controlled release behavior in response to physiological ionic strength. Dense alginate networks delayed ion exchange and prolonged structural integrity, while elevated external ionic conditions triggered rapid disintegration and immediate payload release. This simple and scalable system offers a biocompatible platform for the oral delivery of lipophilic active compounds without the need for surfactants or complex fabrication steps.

## 1. Introduction

Oral administration remains the most preferred route for drug delivery owing to its convenience, patient compliance, and cost-effectiveness [1,2]. However, for hydrophobic compounds, limited aqueous solubility leads to poor absorption in the gastrointestinal (GI) tract, posing significant challenges to effective oral delivery [3,4,5]. To overcome the poor water solubility of hydrophobic compounds, oil-in-water (O/W) emulsions are widely employed using surfactants, lipids, or polymer-based carriers to improve solubility and enhance oral bioavailability [6,7,8,9]. Nevertheless, because emulsions generally rely on surfactants to achieve long-term stability, their use can lead to concerns regarding mucosal irritation, toxicity, and regulatory limitations [10,11,12]. To overcome these limitations, recent efforts have focused on developing surfactant-free encapsulation platforms that can simultaneously ensure structural stability and enable effective release of hydrophobic oil-based compounds under physiologically relevant conditions [13,14,15].

Alginate-based hydrogels have been extensively studied in this context due to their biocompatibility, ionic gelation capability, and tunable mechanical properties [16,17,18,19]. When crosslinked with divalent cations such as Ca^2+^, alginate rapidly forms a robust hydrogel network. The network structure can be tuned by adjusting the polymer concentration and crosslinking conditions, which, in turn, modulates the hydrogel morphology and release kinetics. Despite these advantages, conventional fabrication methods, including extrusion and microfluidics, face technical challenges when handling high-viscosity fluids such as alginate solutions [20,21]. In particular, microfluidic devices struggle to maintain stable flow and droplet formation in the presence of crosslinking agents, which can lead to premature gelation and reduced reproducibility [22,23,24,25]. As an alternative, electrospraying has emerged as a promising technique that enables the handling of high-viscosity fluids and facilitates the formation of monodisperse droplets [26,27,28]. Electrospraying enables the generation of monodisperse droplets across a broad viscosity range without the need for surfactants while allowing direct injection of the fluid into an ionic crosslinking bath to induce gelation. This process simplifies bead production under diverse fluid conditions and facilitates scalability for large-scale manufacturing.

**Scheme 1:**
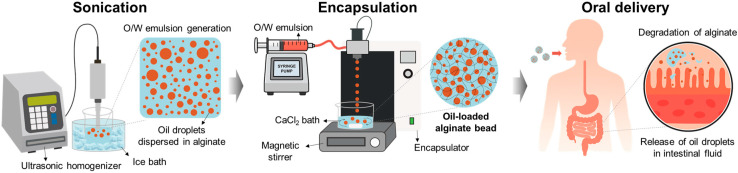
Schematic illustration of the electrospray-based fabrication and oral delivery of matrix-stabilized alginate hydrogel beads encapsulating hydrophobic oil droplets.

In this study, we present a matrix-stabilized, surfactant-free encapsulation strategy for hydrophobic oil-based compounds using a single-step electrospraying process. Oil-in-water dispersions are prepared by ultrasonication of hydrophobic oils into alginate solutions of varying viscosities, enabling kinetic stabilization without the use of surfactants or complex fabrication procedures. The resulting mixtures are directly electrosprayed into calcium chloride baths to form uniform alginate hydrogel beads with embedded oil droplets. We demonstrate that increasing the alginate concentration enhances solution viscosity, which, in turn, suppresses phase separation, improves dispersion stability, and ensures superior bead sphericity and morphological uniformity. Furthermore, the resulting hydrogel beads exhibit pH- and ion-responsive degradation behavior under physiologically relevant conditions, enabling controlled release of the encapsulated hydrophobic compounds. Both alginate and isopropyl myristate used in this system are well-known biocompatible materials, previously validated in a variety of biomedical and oral delivery applications. This approach offers a scalable, biocompatible oral delivery platform for lipophilic active ingredients, achieved without the need for surfactants or multi-step processing. A schematic overview of the entire formulation process is provided in Scheme 1.

## 2. Materials and Methods

### 2.1. Materials

Sodium alginate (Alginic acid sodium salt, (Mw 120,000–190,000 g mol^−1^, M/G ratio = 1.56) was purchased from Sigma-Aldrich (Saint Louis, MO, USA). Isopropyl myristate (IPM, ≥95%, Lot# 5038-4400) was obtained from Daejung Chemicals (Siheung, Republic of Korea), and Oil Red O (Lot# SLBR6841V) was obtained from Sigma-Aldrich. Calcium chloride anhydrous (CaCl_2_, ≥93%, Lot# A0425202) was purchased from ACROS Organics (Geel, Belgium). Phosphate-buffered saline (PBS, 10×, without calcium and magnesium, Cat# SH30258.01) was purchased from Cytiva (Logan, UT, USA). Deionized (DI) water (EXL^®^ 18.2 MΩ∙cm at 28 °C) was produced using a purification system (PURE RO15, PURETECH Co., Ltd., Seongnam, Republic of Korea) and used for all aqueous solutions. All chemicals were used without further purification.

### 2.2. Ultrasonication

O/W emulsions were prepared by dispersing 1% (*v*/*v*) IPM containing Oil Red O (0.01% *w*/*v*) into sodium alginate solutions of varying concentrations (1%, 2%, and 3% *w*/*v*) without surfactants. A 500 W probe-type sonicator (VCX 500, Sonics and Materials, Newtown, CT, USA) was used with a 13 mm probe tip. The emulsification was carried out in a 100 mL total volume using an 80% duty cycle (5 s on/5 s off) for 15 min, maintaining the emulsion temperature at 4 °C using an ice bath, resulting in a total process duration of 30 min. Detailed experimental conditions are summarized in Table 1.

### 2.3. Alginate Hydrogel Bead Formation via Electrospraying

The emulsified alginate mixtures were processed using a BÜCHI B-390 encapsulator (Büchi, Flawil, Switzerland) equipped with a 1000 µm nozzle to generate hydrogel beads. A fixed vibration frequency of 100 Hz was applied, and flow rates were varied between 0.5 and 9 mL/min depending on experimental conditions. This range corresponds to the typical dripping mode of the device, which allows stable droplet formation using high-viscosity alginate dispersions. The selected range was optimal for maintaining production stability while enabling morphological comparison across conditions. The droplets were collected in 100 mM CaCl_2_ solution to induce ionic crosslinking and gelation. The resulting beads were washed three times with deionized water and stabilized at room temperature for 30 min prior to further analysis. Each washing step (~1–2 min) aimed to remove residual calcium ions and unreacted materials. Minor swelling may have occurred due to osmotic shift from the CaCl_2_ solution to deionized water, but no noticeable morphological change was observed. The 30 min stabilization step allowed surface equilibration prior to analysis, which was performed on the same day under hydrated conditions. Detailed experimental conditions are summarized in Table 2.

### 2.4. Microscopy and Morphological Analysis

The morphology of the alginate beads was observed using a stereo microscope (LEICA S9i, Leica Microsystems, Wetzlar, Germany) equipped with an integrated camera. Bead shape was analyzed using ImageJ software (version 1.53t). Circularity was calculated using the following Formula (1):

(1) $$Circularity=\frac{4\pi \;\times \;Area}{{\left(Perimeter\right)}^{2}}$$

Values close to 1.0 indicate a near-perfect sphere. The aspect ratio (AR) was defined as the ratio of the major axis to the minor axis of each bead:

(2) $$AR=\frac{Major\;axis}{Minor\;axis}$$

For each condition, at least 30 beads were analyzed, and the results were reported as mean ± standard deviation.

## 3. Results

### 3.1. Preparation of Oil Dispersion via High-Viscosity Alginate Solution

**Figure 1:**
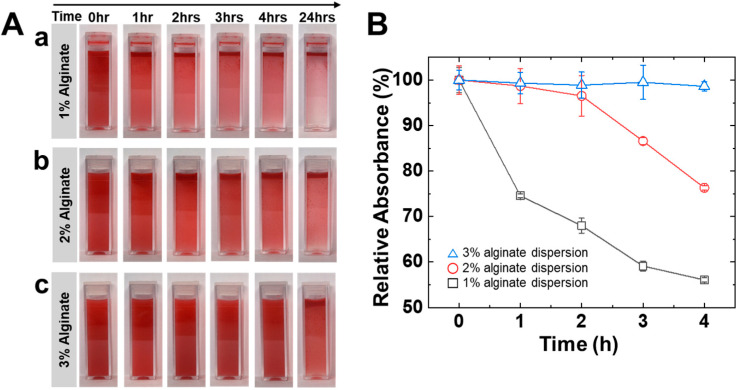
Preparation of oil dispersion in high-viscosity alginate solution (**A**) Time-lapse images of oil dispersions containing different alginate concentrations: (**a**) 1%, (**b**) 2%, and (**c**) 3% (*w*/*v*). (**B**) Relative absorbance at 520 nm measured at the bottom of the cuvettes, normalized to the initial value (A_0_), representing dispersion stability over time. The data for 1%, 2%, and 3% alginate are represented by black squares, red circles, and blue triangles, respectively.

Oil-in-water (O/W) dispersions are prepared by dispersing 1% (*v*/*v*) isopropyl myristate (IPM) into alginate aqueous solutions (1%, 2%, and 3% *w*/*v*) via ultrasonication, without the use of surfactants. The detailed preparation conditions are shown in Table 1. To visualize phase separation, Oil Red O dye was added to the oil phase, and the O/W dispersions containing different concentrations of alginate were monitored over 24 h, as shown in Figure 1A.

As shown in Figure 1A(a), the dispersion containing 1% alginate exhibits rapid phase separation within 1 h, followed by distinct creaming after 3 h. The dispersion containing 2% alginate remains relatively stable during the first hour but begins to exhibit gradual phase separation after 3 h (Figure 1A(b)). In contrast, the dispersion containing 3% alginate maintains visual homogeneity for at least 4 h, indicating enhanced stability at higher alginate concentrations (Figure 1A(c)). The enhanced stability of the dispersions originates from the increased viscosity at higher alginate concentrations, which reduces the mobility of oil droplets and thereby delays coalescence and creaming [29]. This restriction of motion helps maintain visual homogeneity by limiting droplet aggregation over time. High-viscosity matrices reduce the mobility of droplets, thereby minimizing their coalescence and migration. To quantitatively assess dispersion stability, we measured the absorbance of each sample at 520 nm using a UV–visible spectrophotometer (GENESYS 180, Thermo Scientific, Waltham, MA, USA). Oil Red O dye was incorporated into the oil phase to enable optical detection of dispersed droplets. Since oil droplets gradually rise to the top over time due to creaming, the lower portion of the sample becomes increasingly transparent. Therefore, by monitoring the absorbance at the bottom of the cuvettes, we could indirectly assess the degree of droplet retention in the dispersed state. The samples used for this measurement were identical to those shown in Figure 1A, prepared with 1%, 2%, and 3% (*w*/*v*) alginate under the same emulsification conditions. The absorbance values were normalized to the initial value (A_0_) and expressed as percentages to represent the relative amount of oil droplets remaining in the lower region over time. As shown in Figure 1B, the 1% alginate sample exhibited a rapid decrease in absorbance, indicating significant droplet migration to the upper layer. In contrast, the 3% alginate sample maintained over 95% of its initial absorbance even after 4 h, confirming enhanced dispersion stability at higher alginate concentrations. After 12 h, however, visible phase separation occurs in all conditions, and thus all further experiments are conducted within 1 h of dispersion preparation. In conclusion, increasing the alginate concentration leads to the formation of a physical matrix that improves both the structural homogeneity and kinetic stability of O/W emulsions without the need for surfactants.

### 3.2. Electrospray-Assisted Fabrication of Uniform Alginate Hydrogel Beads

We investigate how flow rate and alginate viscosity influence the formation of uniform alginate hydrogel beads. Oil-in-water dispersions prepared using 1%, 2%, and 3% (*w*/*v*) alginate solutions were electrosprayed into a 0.1 M calcium bath under identical conditions, except for flow rate and alginate concentration. The detailed electrospraying parameters are listed in Table 2. Hydrogel bead formation was achieved across all alginate concentrations; however, distinct differences were observed in structural integrity and morphological uniformity. Beads formed from the 1% alginate dispersion exhibited irregular and flattened morphologies. The generated droplets displayed elongated tails and unstable shapes, which are attributed to deformation during free fall due to insufficient viscosity. The low-viscosity matrix lacks sufficient elastic resistance to maintain surface tension during flight, causing the droplets to collapse or spread upon impact with the calcium bath. As a result, the 1% alginate dispersion failed to produce spherical beads and was excluded from quantitative analysis. To further evaluate the effect of viscosity on bead morphology, beads were generated from 2% and 3% alginate dispersions under various flow rates ranging from 0.5 to 9 mL/min.

**Figure 2:**
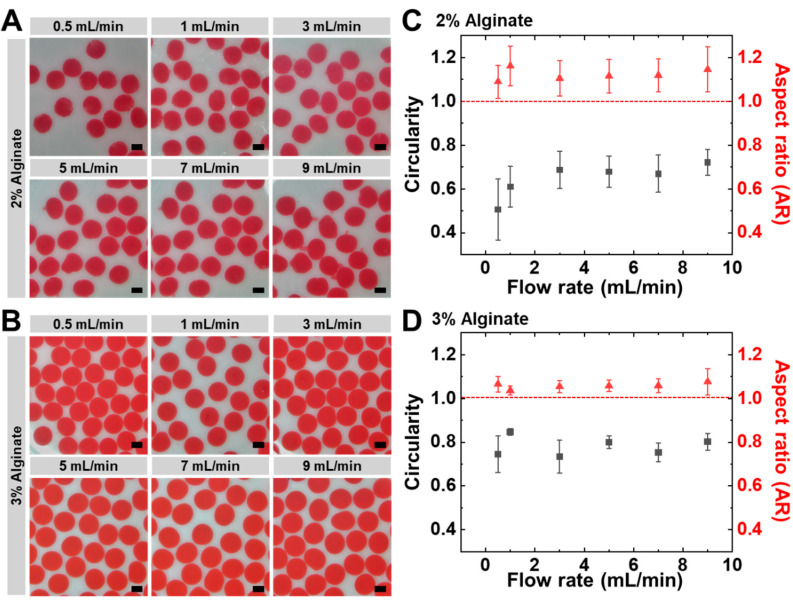
Formation of uniform alginate hydrogel beads via electrospraying. (**A**,**B**) Representative optical images of beads generated from 2% (**A**) and 3% (**B**) alginate solutions under varying flow rates (0.5–9 mL/min). (**C**,**D**) Quantitative analysis of bead circularity (black) and aspect ratio (red). Data are presented as mean ± standard deviation (*n* = 30). All scale bars represent 1 mm.

As shown in Figure 2A, the circularity of beads generated from the 2% alginate dispersion begins to decrease gradually, even below 5 mL/min. However, morphological deformation becomes visually evident at flow rates exceeding 5 mL/min, with distinctly elongated and elliptical shapes observed above 7 mL/min. This deformation is likely due to increased inertial and shear forces at higher flow rates, which exceed the mechanical resistance provided by the 2% alginate solution. Although 2% alginate exhibits moderate viscosity, it may not be sufficient to fully suppress droplet elongation during flight and impact, leading to distortion of bead shape. In contrast, the 3% alginate dispersion produces nearly spherical beads with smooth and uniform surfaces, suggesting that the high viscosity plays a critical role in maintaining droplet integrity throughout the electrospraying and gelation processes (Figure 2B). Quantitative analysis of circularity and aspect ratio (AR) supports these observations (Figure 2C,D). As shown in Figure 2C, the 2% alginate group exhibits a decrease in circularity and an increase in aspect ratio (AR) with an increasing flow rate, indicating morphological deformation. The 3% alginate group maintains high circularity and an almost ideal aspect ratio (~1.0) across all flow rates, indicating superior morphological stability (Figure 2D). In addition, the markedly reduced error bars compared to the 2% group confirm the high reproducibility of bead formation under this condition. The high reproducibility observed under 3% alginate conditions reflects the specific properties of the alginate used in this study. Since viscosity and gelation behavior vary with alginate molecular weight and M/G ratio, reproducibility may differ when using alginates with different compositions. These results suggest that increasing alginate concentration enhances solution viscosity, thereby providing greater mechanical resistance to shear-induced deformation during electrospraying. Collectively, increasing viscosity enhances resistance to deformation by strengthening interfacial tension and regulating droplet breakup under electric fields. These results underscore the importance of alginate viscosity in forming structurally stable and morphologically uniform beads under variable electrospraying conditions, supporting its potential for downstream applications.

### 3.3. Degradation-Driven Triggered Release from Tunable Alginate Hydrogel Networks

**Figure 3:**
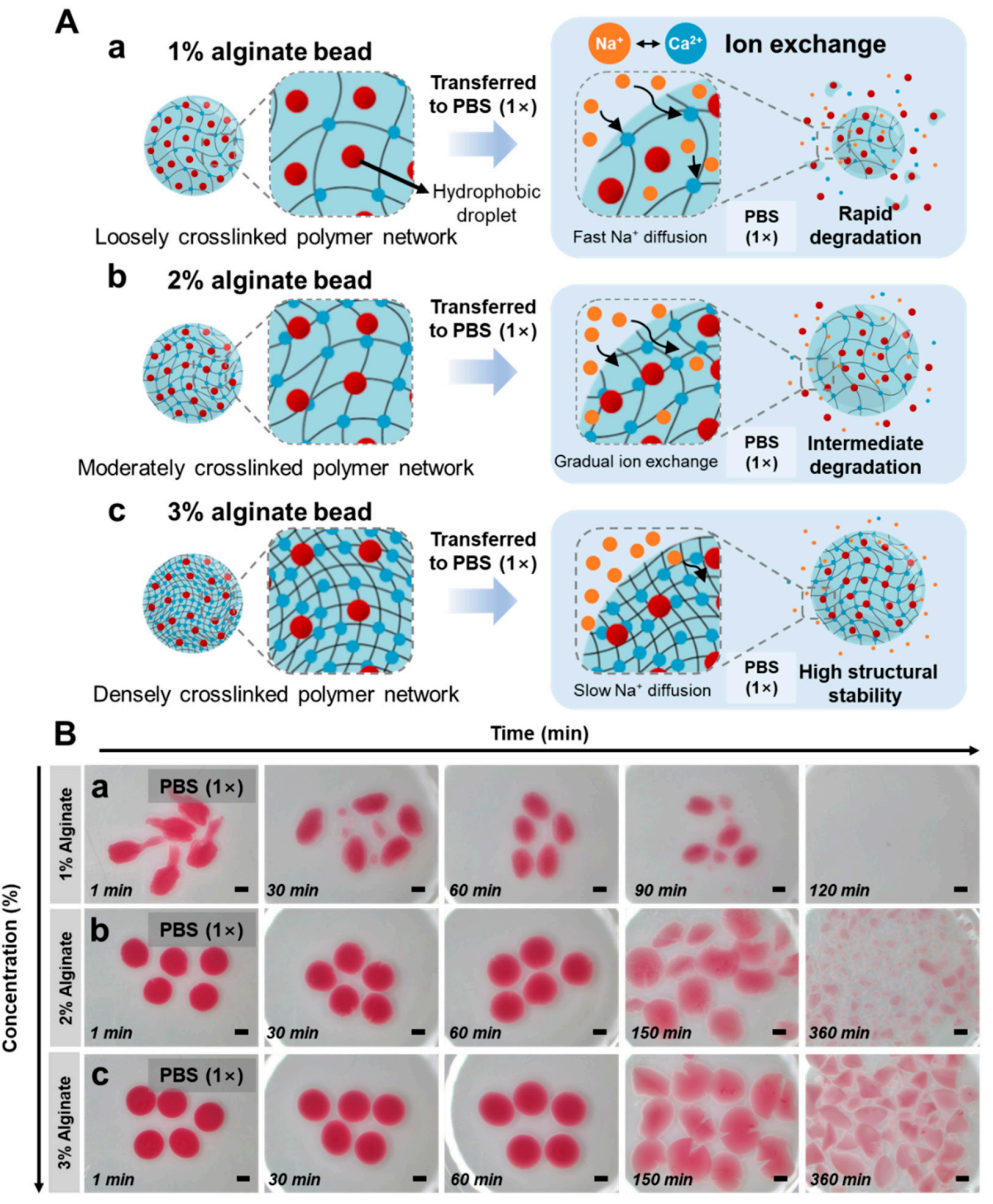
Controlled release through tunable alginate hydrogel networks. (**A**) Schematic illustration of ion exchange between Na^+^ in PBS and Ca^2+^ in alginate beads with varying concentrations: (**a**) 1%, (**b**) 2%, and (**c**) 3% alginate. (**B**) Time-lapse optical images of beads in 1× PBS (pH 7.4): (**a**) 1%, (**b**) 2%, and (**c**) 3% alginate. The red color indicates the presence of Oil Red O-labeled oil droplets. All scale bars represent 1 mm.

Alginate-based hydrogel beads enable modulation of the crosslinked network by adjusting alginate concentration. To evaluate how network formation and degradation behavior vary with alginate content, beads prepared with 1%, 2%, and 3% (*w*/*v*) alginate were dispersed in phosphate-buffered saline (PBS, 1×, pH 7.4) and observed over time. PBS promotes ion exchange between sodium ions (Na^+^) and calcium ions (Ca^2+^) in the alginate network, leading to gradual degradation of the hydrogel structure. Its 1× concentration and pH 7.4 closely mimic the physiological ionic strength and mildly alkaline environment of the small intestine. The schematic in Figure 3A illustrates how ion exchange and pH contribute to the degradation behavior of alginate beads with hydrogel networks formed at different alginate concentrations. Beads fabricated with 1% alginate formed a relatively loose polymer network (Figure 3A(a)). Such a low-density structure facilitates rapid penetration of Na^+^ ions into the network, resulting in the displacement of crosslinked Ca^2+^ ions. As Ca^2+^ ions are replaced, the network weakens significantly, thereby accelerating structural degradation. In contrast, higher alginate concentrations (2% and 3%) result in more densely crosslinked networks that contain a greater number of Ca^2+^ binding sites, thereby requiring more extensive ion exchange for complete degradation. This denser matrix also limits ion mobility, contributing to the overall delay in Na^+^-mediated disintegration. As a result, the structural integrity of the beads is enhanced, leading to slower degradation of the hydrogel matrix and consequently delayed release of the encapsulated oil droplets (Figure 3A(b,c)). As shown in Figure 3B, optical images were captured over time to monitor the structural changes in beads with different network densities after immersion in PBS. Beads prepared from 1% alginate dispersion rapidly disintegrate without noticeable swelling and lose their structural integrity within 90 min (Figure 3B(a)). Beads prepared from 2% alginate dispersion exhibit moderate swelling within the first 60 min, followed by gradual network relaxation and fragmentation between 150 and 360 min (Figure 3B(b)). Meanwhile, beads prepared from 3% alginate dispersion exhibit gradual swelling and retain their overall structural integrity throughout the observation period, displaying the slowest degradation among all tested concentrations (Figure 3B(c)). These differences are attributed to the increased gel density associated with higher alginate concentrations, which hinders Na^+^ diffusion and delays the ion exchange process, ultimately postponing the onset of bead degradation [30]. The ability to finely tune the degradation behavior by modulating the alginate network suggests that this formulation strategy holds strong potential for achieving stimuli-responsive triggered release of hydrophobic compounds under physiological conditions.

### 3.4. Stimuli-Responsive Degradation and Release from Alginate Hydrogel Beads Triggered by Ionic Environment

**Figure 4:**
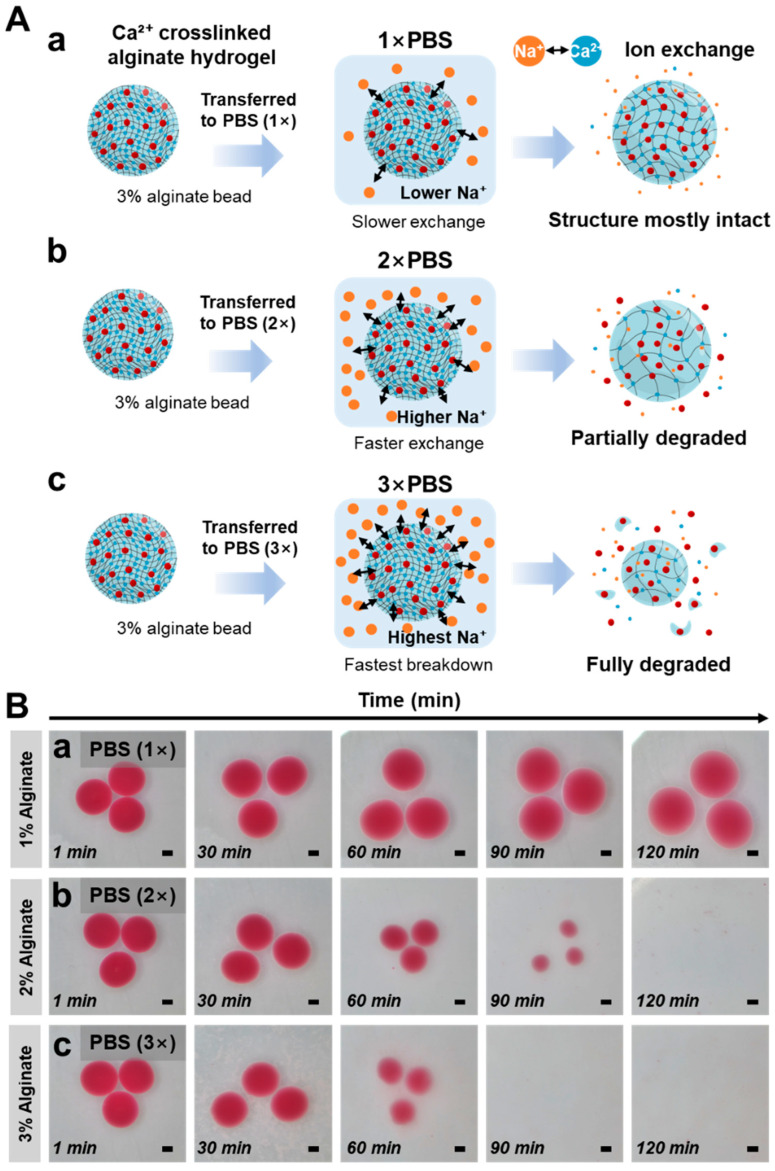
Controlled release from alginate hydrogel beads under ionic environments. (**A**) Schematic illustration of the ion exchange mechanism for beads composed of (**a**) 1%, (**b**) 2%, and (**c**) 3% alginate. As the Na^+^ concentration in PBS increases (1×, 2×, 3×), the displacement of Ca^2+^ ions from the alginate matrix is promoted, accelerating structural degradation. Arrows indicate the direction of Na^+^ influx, and red dots represent oil droplets stained with Oil Red O. (**B**) Time-lapse optical images showing degradation of beads corresponding to (**a**) 1%, (**b**) 2%, and (**c**) 3% alginate under the same ionic conditions. All scale bars represent 1 mm.

To evaluate how external stimuli affect the degradation and release behavior of alginate hydrogel beads, we focused on changes in ionic strength as a representative trigger. Ionic strength directly influences the ion exchange between Na^+^ and Ca^2+^ within the alginate network, thereby modulating the structural integrity and disintegration rate of the beads. We investigated the effect of ionic strength by immersing alginate beads prepared from 3% (*w*/*v*) alginate solution into phosphate-buffered saline (PBS) solutions with increasing ionic strengths (1×, 2×, and 3×). The 1× PBS condition approximates the physiological ionic strength of the intestinal lumen, whereas 2× and 3× PBS represent high-salt environments designed to evaluate how increased ionic gradients affect Ca^2+^ displacement and hydrogel destabilization. As illustrated in Figure 4A, increasing Na^+^ concentration accelerates the inward diffusion of monovalent cations and promotes the displacement of Ca^2+^ ions involved in crosslinking within the alginate network. Under the 1× PBS condition (Figure 4A(a)), the relatively mild Na^+^ gradient leads to a gradual ion exchange, resulting in a transient swelling prior to major degradation. In contrast, beads exposed to 2× and 3× PBS experience a steeper ionic gradient, which accelerates Ca^2+^ displacement and induces immediate structural collapse without swelling (Figure 4A(b,c)). To visually assess the effect of ionic strength on bead degradation, optical microscopy was performed over a 120 min period under each condition (Figure 4B). Under the 1× PBS condition, the beads gradually swell while maintaining structural integrity, and no visible degradation occurs during the observation period (Figure 4B(a)). In 2× PBS, partial degradation is observed within 60 min, and complete degradation occurs between 90 and 120 min, leaving no visible structure (Figure 4B(b)).

In 3× PBS, evident structural collapse is observed within 30 min, and near-complete degradation is confirmed by 90 min (Figure 4B(c)). These results suggest that external ionic strength serves as a key determinant of the structural stability of alginate hydrogel beads. The degradation of alginate gels is primarily induced by ion exchange between monovalent and divalent cations, where Na^+^ ions replace crosslinked Ca^2+^ ions, thereby weakening the network and leading to structural collapse [30].

Under low ionic strength conditions, the ion exchange proceeds slowly, allowing gradual ion penetration and resulting in observable swelling. In contrast, under high ionic strength conditions, rapid Ca^2+^ displacement occurs, triggering immediate structural collapse without prior swelling [31]. Taken together, these findings suggest that adjusting external ionic strength offers a viable strategy to modulate the release rate of encapsulated hydrophobic compounds.

## 4. Conclusions

This study established a matrix-based physical encapsulation strategy for embedding hydrophobic compounds within alginate hydrogel beads using electrospraying. Surfactant-free oil-in-water dispersions were prepared via ultrasonication into high-viscosity alginate matrices, in which droplet coalescence and phase separation were suppressed by enhanced kinetic stabilization. Electrospraying these dispersions into calcium chloride solution enabled the formation of monodisperse beads with tunable morphology; higher alginate concentrations improved sphericity and maintained bead uniformity across variable flow conditions. Beads with denser alginate networks exhibited slower ion exchange and sustained structural integrity, resulting in prolonged retention and delayed release of encapsulated oil droplets under physiological conditions. Furthermore, increasing the external ionic strength triggered rapid gel degradation and immediate droplet release, confirming the ion-responsiveness of the system. Altogether, this physically stabilized, and stimuli-responsive platform demonstrated high potential for the oral delivery of lipophilic active compounds and could be applied to the development of pharmaceutical, nutraceutical, or postbiotic formulations requiring controlled release behavior under physiological conditions.

## Figures and Tables

**Table 1:** Ultrasonication conditions for surfactant-free preparation of alginate–IPM dispersions.

Alginate Concentration (% *w*/*v*)	Oil Content (% *v*/*v*)	Surfactant	Total Volume (mL)	Sonication Power (W)	Cycle (%)	Sonication Time (min)	Actual Duration (min)
1	1	None	100	500	80	15	30
2	1	None	100	500	80	15	30
3	1	None	100	500	80	15	30

**Table 2:** Electrospraying conditions and morphological characteristics of alginate beads at varying concentrations.

Alginate (%)	Nozzle (μm)	Voltage (kV)	Frequency (Hz)	Flow Rate (mL/min)	Bead Shape
1	1000	1.0	100	0.5~9	Irregular
2	1000	1.0	100	0.5~9	Spherical
3	1000	1.0	100	0.5~9	Spherical

## Data Availability

Data are contained within the article.
